# A Comprehensive Review of Medicinal Leeches in Plastic and Reconstructive Surgery

**DOI:** 10.1097/GOX.0000000000002555

**Published:** 2019-12-26

**Authors:** Paige N. Hackenberger, Jeffrey E. Janis

**Affiliations:** From the * The Ohio State University College of Medicine, Columbus, Ohio; † Department of Plastic Surgery, The Ohio State University Wexner Medical Center, Columbus, Ohio.

## Abstract

Medicinal leeches are a US Food and Drug Administration-approved treatment for venous congestion in graft tissue to promote healing and can serve as a nonsurgical option for plastic surgery patients with concern for tissue compromise. Although there is a wealth of documentation on medicinal leech therapy, the surgical space currently lacks an updated summary of proper indications, use, and risks as they pertain to plastic surgical patients. The purpose of this article is to provide a platform for understanding the recent literature as it relates to reconstruction to improve understanding of indications and necessary considerations in using hirudotherapy. Topics examined include basics of hirudotherapy, indications in plastic surgery, implementation (leech application, number and duration of therapy, and removal), risks (infection and bleeding), and alternative treatments. The evidence provided will aid in physician understanding and implementation, patient counseling, and the informed consent process.

## BACKGROUND

Hirudotherapy, the use of leeches for medicinal purposes, dates back to ancient medicinal practices of various cultures as a method of balancing “biological humors” believed to contribute to poor health.^1–4^ Since that time, a developing understanding of the mechanisms of action underlying the therapeutic advantages has expanded the scope of hirudotherapy to include treatment of osteoarthritis, autoimmune disease, cardiovascular disease, cancer, and complications of diabetes.^1,2,5^ The approval of leeches as a medical device in 2004 by the US Food and Drug Administration (FDA) further supports the expanding understanding around practical roles of medicinal leeches.^6^

**Fig. 1.**
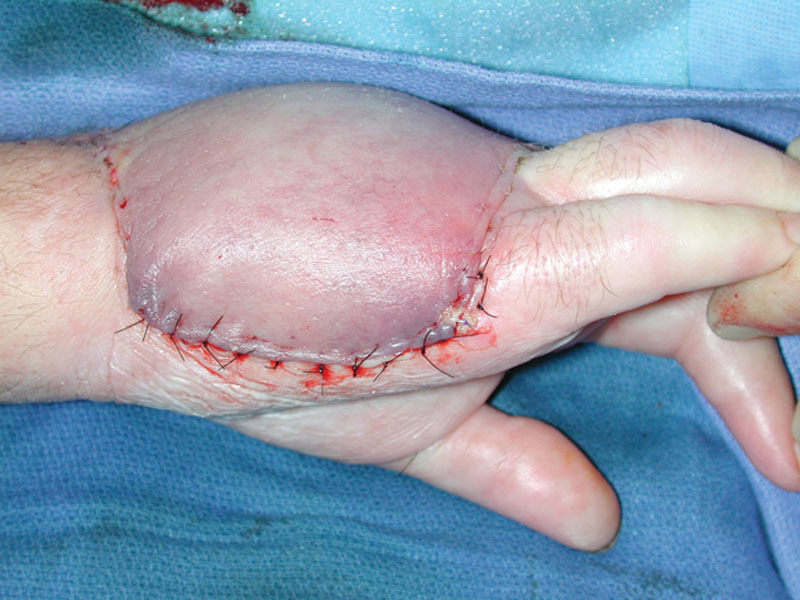
Local flap indicating signs of venous congestion.

Within plastic and reconstructive surgery, the predominant role of leeches is to relieve venous congestion in compromised flaps. In the postoperative period, flap success is reliant on effective monitoring for vascular compromise, with venous thrombosis described as both the most likely and quickly damaging event.^7^ Clinical signs of venous congestion include tissue with cyanotic/dusky coloration, increased turgor, cool tactile temperature, brisk capillary refill, and rapid dermal bleeding (Fig. 1).^8,9^

Although much is understood about medicinal leeches, the paucity of randomized control trials and evolving risk–benefit profile, including the emergence of leech-associated multidrug-resistant infectious organisms, has led to both a lack of education and confidence among plastic surgeons and trainees as it pertains to incorporating hirudotherapy into postoperative treatment decision sets. This in turn precludes adequate counseling for patients in cases which may benefit from medical leech therapy as a salvage strategy when first-line treatments and interventions are not successful.

## MECHANISM OF ACTION

Leech therapy works by simultaneously alleviating tissue capillary pressure and promoting local anticoagulation through 3 general mechanisms of action: active suction, secretion of vasoactive substances such as hirudin, and passive oozing of blood through bite wounds after leech removal.

The salivary glands of leeches emit many biologically active substances with anticoagulant, thrombin regulatory, anti-inflammatory, analgesic, platelet inhibitory, extracellular matrix degradative, and antimicrobial properties (Table 1). The most common and well studied of these substances is the natural thrombin inhibitor, hirudin.^1,10,11^ Recent studies by Müller et al have developed a greater understanding of unique variations in components of leech saliva, termed hirudin-like factors, which promote local anticoagulation in the surrounding tissues and contribute to ongoing blood removal and thrombolysis.^10,12^

**Table 1.** Biologically Active Substances in Leech Saliva^2,11^

Mechanism of Action	Substances
Anticoagulation and thrombin regulatory	Hirudin, bufrudin, gelin, lefaxin, destabilase, new leech protein-1, whitide, whitmanin
Anti-inflammatory and analgesic	Antistasis, hirustasin, ghilatens, eglin C, leech-derived tryptase inhibitor, complement C1 inhibitor, guamerin, piguamerin, carboxypeptidase inhibitor, bdellins, bdellastasin
Platelet inhibition	Saratin, calin, apyrase, decorsin
Extracellular matrix degradation	Hyaluronidase, collagenase
Antimicrobial	Destabilase, chloromycetyn theromacin, theromyzin, peptide B

## APPLICATIONS TO PLASTIC SURGERY

Prevention of flap necrosis is the most common indication for hirudotherapy in plastic surgery; however, the use of leeches for replantation of digits, lips, ears, nipples, nasal tips, and penises have all been documented.^2,9,13–16^ Flap necrosis secondary to venous congestion can progress within a mere 3 hours, and success of leeching depends on provision of sufficient temporary venous outflow while neovascularization of the flap occurs.^17–19^

**Fig. 2.**
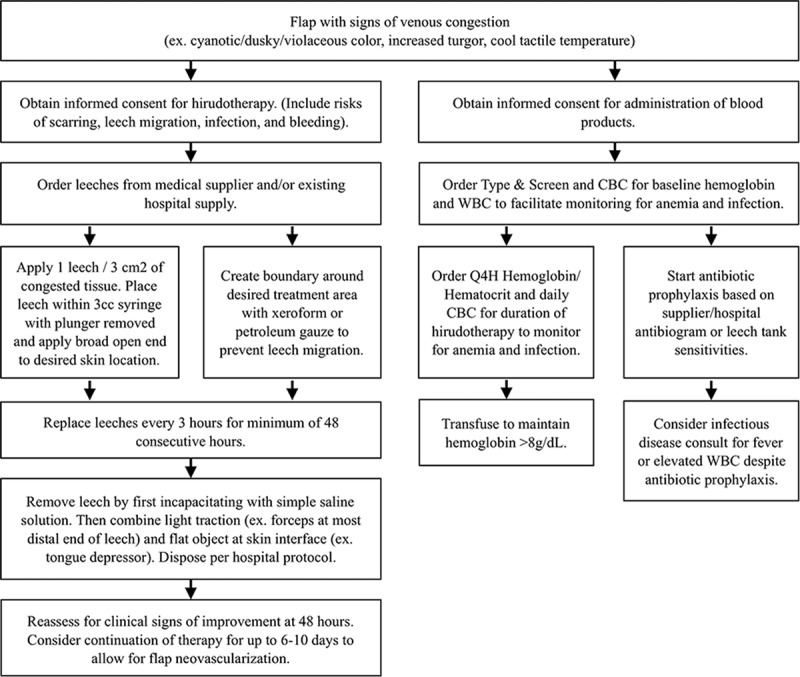
Recommended best practices for leech therapy on flaps with venous congestion. CBC, complete blood count; Q4H, every four hours; WBC, white blood cell count.

In the most recent systematic review of the success of hirudotherapy in plastic and reconstructive surgery, Whitaker et al demonstrated a total tissue salvage rate of 78%, with only 22% of tissues treated with medical leeches ultimately requiring resection.^13^ Other studies indicate a slightly lower rate of salvage at 60.9%, considering success as no flap loss or partial flap loss not requiring further reconstructive procedures.^14^ Figure 2 shows the best practices from the following information.

## IMPLEMENTATION

With the increasing evidence to support the use of leeches for the treatment of venous congestion in plastic surgery patients, there remains controversy over the method of application, timing, and duration of treatment necessary to support optimal outcomes for tissue survival. Additionally, institutional logistics must be addressed to ensure proper storage and monitoring of the leeches as well as adequate training and counseling of nursing staff.^20^

### Application

Leeches should be placed in areas of clinically significant venous congestion, which are demarcated by deep cyanosis or violaceous color of the tissue. Leeches attach to their human host via a large caudal sucker and create a Y-shaped bite in the skin via a smaller cephalic sucker during which an anesthetic compound is released, often making the bite painless.^1,9,21^

Various methods have been described for leech application. Traditionally, they are applied using a 3-mL syringe with the plunger removed, placing the open end over the compromised tissue and waiting until the leech securely attaches to the patient’s skin in the desired treatment area.^22,23^ However, this method does not protect against leech migration which often occurs after consuming a complete bloodmeal.^24^ To prevent this, other methods include the placement of leeches within an aerated plastic cup with a hole cut in the bottom, surrounding the leech with a boundary such as Xeroform or petroleum gauze (which leeches avoid), or even suturing the leech body to the patient.^24–27^

### Number and Duration

**Fig. 3.**
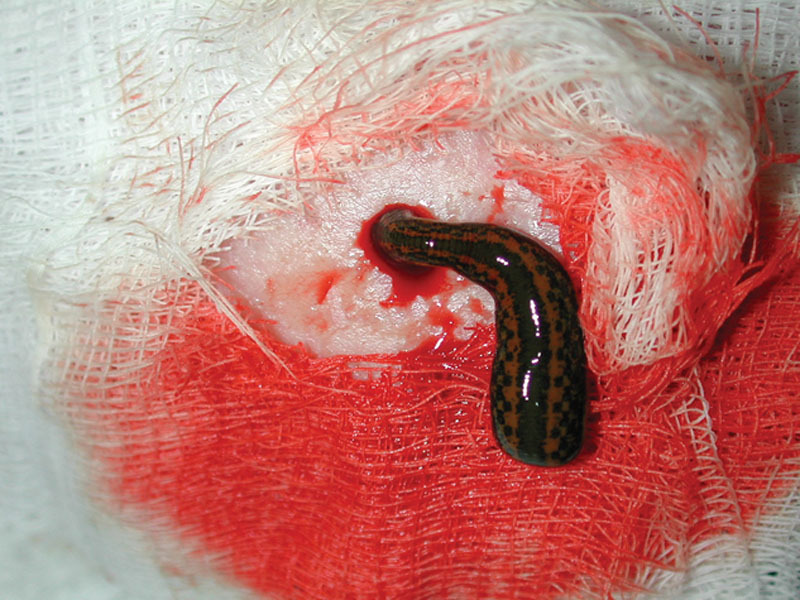
Leech attached to flap with venous congestion.

There exists great variation regarding the number of leeches to apply to congested tissue. This is further complicated by the total area and flap size to be treated. Each applied leech can be expected to consume 5–15 mL of blood during a feeding which may last from 30 to 90 minutes (Fig. 3).^11,24,28,29^ Rothenberger et al observed the most significant improvements in tissue perfusion occur 1 hour after leech application and return to the prehirudotherapy perfusion state by 3 hours after leech application.^15^ This was determined through the use of the oxygen to see (O2C) device which assesses blood flow, relative hemoglobin, and oxygen saturation in tissues and may be useful to specifically guide leech therapy decisions on an individual basis, while being less invasive than fluorescein injections which have been used historically.^15,30^

Published studies range considerably in numbers of leeches applied, ranging from 1 to 5 per treatment, to an area-dependent calculation of 1 leech per 3 cm^2^; similar variation pertains to frequency of leech application from hourly to daily.^11,19,29^ Leech therapy is ideally continued until flap neovascularization, requiring an average of 6–10 days, with an average duration of 4.6 days.^1,14,31^ However, signs of clinical improvement may warrant removal after 48 hours with close monitoring for the need for reapplication based on evolving individual clinical examination.^29,32^

### Removal

It is important during the removal of medicinal leeches to prevent regurgitation of leech digestive tract contents into the wound, as this can lead to infectious complications. Proper removal of leeches should aim to first incapacitate the leech (commonly with a simple saltwater solution) and then combine gentle traction and a flat object to break suction with the skin attachment (eg, tongue depressor).^33^ Leeches without size increase or visible gut peristalsis within 30 minutes after application are likely inactive and should be removed and replaced to facilitate the best outcome.^29^

**Fig. 4.**
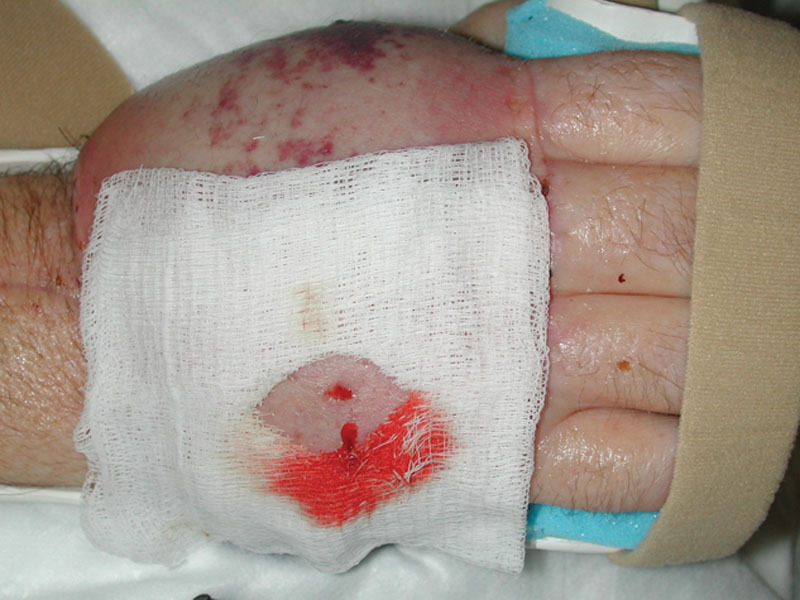
Leech bite wound with passive oozing.

As part of their therapeutic purpose, bite wounds are expected to passively ooze for up to 24 hours after leech removal (Fig. 4); however, bite sites with ongoing bleeding or anemia requiring large transfusion volume can be primarily sutured to promote hemostasis.^29,34^

## RISKS

There are several contraindications as well as complications associated with hirudotherapy treatment. Providers should be familiar with absolute and relative contraindications to treatment with medicinal leeches (Table 2).

**Table 2.** Contraindications to Medicinal Leech Therapy^34,35^

Comorbid conditions	Anemia
	Hematologic malignancy
	Hemophilia
	Hemorrhagic diathesis
	HIV infection
	Mixed arteriovenous insufficiency
	Unstable medical status
	Cachexia
	Decompensated hepatobiliary disease
	Hypotension
	Sepsis
Medications	Anticoagulant
	Immunosuppressant
	Other vasoactive
Individual factors	Leech allergy
	Leech intolerance
	Pregnancy/lactation
	Propensity for keloid scar formation
	Refusal of blood transfusion

The overall complication rate with hirudotherapy is 21.8%.^13^ These vary in likelihood and severity, with localized itching at the bite being the most common (37%–75%), and infection and bleeding posing the greatest risk.^11,29^ Other complications include regional lymphadenitis with subfebrile temperatures, leech migration to nontherapeutic sites, bite-associated scarring, psychosis, prerenal azotemia, and pain.^13,29^

### Infection

Leeches rely on a symbiotic relationship with *Aeromonas* species bacteria such as *Aeromonas hydrophila* and *Aeromonas veronii* within their digestive tract to process ingested bloodmeal.^36-37^ This symbiotic relationship portends leeches as vectors for infections in humans, which occurs when these digestive bacteria are injected via saliva or regurgitation at therapeutic bite sites. As a result of this risk, up to 79% of patients receiving hirudotherapy are also started on antibiotic prophylaxis.^13^ Whitaker et al demonstrated that 14.4% of patients receiving medicinal leech therapy contracted an associated infection and that these infections significantly reduced the likelihood of tissue salvage (37.4%) compared to patients without an infection (88.3%).^13^ *Aeromonas* species account for 88% of leech-associated infections, but recent reports of several other pathogens, including *Serratia marcescens*, *Vibrio fluvialis*, various viruses, and emerging multidrug-resistant organisms, have increased the risk profile of leech therapy.^13,38–41^ Third-generation cephalosporins, fluoroquinolones, aminoglycosides, tetracycline, and trimethoprim–sulfamethoxazole are the most effective antibiotics against *Aeromonas* species and are first line for infection prophylaxis.^6,42,43^

Due to emergence of resistant organisms, hospitals should conduct regular sampling, culture, and susceptibility indices of leech tank water, which should be used to determine institution-specific chemoprophylaxis measures.^6^ Studies by Litwinowicz and Blaszkowska also suggest prefeeding leeches with appropriate antibiotics as a method by which chemoprophylaxis in patients can be eliminated while still reducing infectious risk to an acceptable level.^42^

### Bleeding

Blood loss during leech therapy can result in iatrogenic anemia necessitating blood transfusion and decreased rates of tissue survival. Whitaker et al demonstrated that 50% of patients receiving hirudotherapy require blood transfusions during treatment, and among patients requiring blood transfusion, tissue salvage rates decline to 82.2% from 91.2%.^13^ The average transfusion requirement for patients undergoing treatment with medicinal leeches ranges from 3 to 6 U of packed red blood cells.^29,44^ To effectively monitor and treat medicinal leech bleeding complications, a complete blood count should be taken before leech treatment and hemoglobin and hematocrit should be checked every 4 hours throughout its duration.^13,29^ Additionally, a type and screen should be kept up-to-date, and patients should be transfused to maintain a hemoglobin above 8g/dL throughout therapy.^13,29^

## ALTERNATIVE TREATMENTS

In an attempt to mitigate the risk profile associated with the application of medicinal leeches, several alternatives including both mechanical and chemical approaches have been trialed. To most clearly match the benefits of hirudotherapy, evidence supports alternatives which satisfy 3 main components: suction, chemical anticoagulation, and a surgically created bleeding wound.^45^ Attempts at creation of devices to replace medicinal leeches date back to the early 1800s, but modern approaches have included anticoagulation via surface irrigation, turbulence irrigation, subcutaneous injection, and disk agitation of heparin.^45^ In pedicled flaps, venous catheterization has been described as a superior alternative to leech therapy, leading to improved outcomes and lower rates of complications.^46^ Many questions remain regarding the best form of chemical anticoagulation, the depth of the surgically created bleeding wound, as well as whether intermittent versus constant suction is required for optimal outcomes.

## CONCLUSIONS

Medicinal leeches are an FDA-approved therapy for the treatment of venous congestion in graft tissue and should be considered for nonoperative tissue salvage in plastic surgery patients. Leeches can provide a sufficient venous outlet until flap neovascularization occurs when applied appropriately. Patients undergoing hirudotherapy should be monitored and treated for severe complications such as infection and anemia. More research is necessary to evaluate the marginal benefit of medicinal leeches in settings of venous congestion and to provide an accurate and current risk–benefit assessment for their use.
